# Associations between parenting styles and depressive symptoms in college students: a sequential mediation model and a preliminary randomized study

**DOI:** 10.3389/fpsyt.2026.1788390

**Published:** 2026-04-30

**Authors:** Feifan Han, Dan Peng

**Affiliations:** Center for Mental Health Education and Counseling, Guizhou Medical University, Guiyang, China

**Keywords:** chain mediation, college students, depressive symptoms, group counseling, interpersonal efficacy, meaning in life, parenting style

## Abstract

**Objective:**

To explore the mechanisms underlying depressive symptoms among college students, develop a targeted intervention based on the proposed model, and provide empirical support for university mental health education.

**Methods:**

This study examined the relationships between positive and negative parenting styles and depressive symptoms among college students, with interpersonal efficacy and meaning in life serving as sequential mediators. Positive parenting included emotional warmth, while negative parenting involved rejection and overprotection. To further assess the feasibility of targeting a key component within this model, the study comprised two sequential sub-studies. In Study 1, 1,262 college students from a university in Guizhou Province completed measures of parenting styles (the short-form Egna Minnen av Barndoms Uppfostran, Chinese version; Cronbach’s α=0.67-0.88), interpersonal efficacy (College Students’ Interpersonal Efficacy Scale,α=0.75), meaning in life (The Meaning in Life Questionnaire, α=0.86), and depression (Center for Epidemiological Survey, Depression Scale, α=0.88). A chain mediation analysis was conducted using Model 6 of the PROCESS macro in SPSS. In Study 2, 35 participants with high depression scores and low interpersonal efficacy were selected from Study 1 and randomly assigned to either an experimental group (n=17, receiving seven weekly group counseling sessions, each lasting approximately 90 minutes) or a control group (n=18, receiving no intervention). After the intervention, 3 participants dropped out from the experimental group and 4 from the control group, resulting in 14 participants in each group for the final analysis. A 3 (Time: pre-intervention, post-intervention, 3-month follow-up) × 2 (Group: experimental, control) two-factor mixed design was employed, and repeated measures ANOVA was used to evaluate the intervention effects.

**Results:**

In Study 1, interpersonal efficacy and meaning in life played significant chain-mediating roles in the relationship between parenting styles and depressive symptoms. In Study 2, a significant main effect of the group on interpersonal efficacy was found, with the intervention group showing significantly higher scores than the control group. A significant group-by-time interaction effect was observed: the intervention group exhibited marked post-intervention gains relative to baseline and the control group, with these gains diminishing but remaining above baseline at the 3-month follow-up. No significant effects of the intervention on depression were found.

**Conclusion:**

The findings support the plausibility of a model in which parenting styles, interpersonal efficacy, meaning in life, and depressive symptoms are associated in college students. In the preliminary randomized study, the intervention was associated with improvement in interpersonal efficacy, but no between-group effect was demonstrated for depressive symptoms. These results should therefore be interpreted cautiously and considered preliminary.

## 1 Introduction

Currently, depressive symptoms among college students are highly prevalent and have been increasing year by year (1). Depression significantly affects students’ academic performance, mental health, and overall quality of life (2). Therefore, it is essential to investigate the mechanisms underlying the development of depression in this population, as such research can provide both theoretical foundations and practical guidance for designing targeted interventions. Depression is a common mood disorder characterized by persistent low mood and anhedonia, often accompanied by reduced energy levels and impairments in cognitive and social functioning (3, 4). Among the multiple factors influencing the onset and progression of depression, the family—as the primary microsystem in individual development—plays a critical role (5). Specifically, negative parenting styles (e.g., rejection, overprotection) have been shown to exert a substantial influence on the development of depressive symptoms (6–8). This represents a core component of these mechanisms. Conversely, positive parenting styles (e.g., emotional warmth) can mitigate depressive symptoms and may further reduce the risk of suicidal behavior (9, 10). These findings provide a key entry point for examining potential pathways to depression.

However, the relationship between parenting styles and depression is not always straightforward. From a resource-based perspective, individuals possess internal psychological resources that enable them to cope with stress and maintain emotional well-being (11). When these resources are compromised, vulnerability to depressive symptoms may increase. Previous research indicates that maladaptive parenting styles are associated with reductions in self-related capacities, including self-efficacy, self-esteem, and adaptive coping (12–15), which in turn elevate the risk of depression.

Given the mediating role of psychological resources, this study focuses on interpersonal efficacy and meaning in life. Interpersonal efficacy refers to an individual’s confidence in their ability to accomplish interpersonal tasks and maintain healthy relationships. It plays a crucial role during adolescence and emerging adulthood, when social adjustment and peer interactions are central (16). Positive parenting has been shown to enhance interpersonal efficacy, whereas maladaptive parenting may hinder it (17). Lower levels of interpersonal functioning and social connectedness are consistently associated with higher depressive symptoms, underscoring the importance of social relationships in mental health (18). Similarly, meaning in life—reflecting an individual’s motivation and cognitive perceptions in seeking life’s significance—has been identified as a core protective factor for psychological well-being (19). A strong sense of life meaning helps individuals maintain a positive mindset in the face of setbacks and promotes better psychological adaptability (20). From a resource-based perspective, interpersonal efficacy can be viewed as a domain-specific social-cognitive resource, while meaning in life represents a broader existential resource (11). As a more proximal resource, interpersonal efficacy may contribute to the development of higher-order existential resources, making the proposed sequential pathway theoretically plausible. Empirical evidence suggests that interpersonal competence significantly predicts meaning in life among Chinese college students (21). When these internal resources are weakened, vulnerability to depressive symptoms may increase. Thus, interpersonal efficacy and meaning in life may serve as critical psychological resources in the relationship between parenting styles and depression.

However, no research to date has tested whether these variables work in sequence. From a developmental standpoint, interpersonal efficacy tends to develop earlier and may create conditions that make meaning in life more accessible later. The present study therefore asks not just whether both are related to depression, but whether they follow this order. Examining the sequence helps clarify how the two resources are connected, and could offer clues about when to target each in depression interventions. This is particularly important for individuals in a depressed state, whose emotional distress often stems from challenges in social relationships (22). If this sequential pathway is supported, the findings would offer valuable insights for depression interventions. Unlike relatively stable parenting styles, interpersonal efficacy is a more accessible and malleable target. From a resource-based perspective, interpersonal efficacy is a more proximal and developmentally earlier resource than meaning in life, making it a potentially more feasible entry point for intervention. Improving interpersonal efficacy may not only alleviate interpersonal difficulties but also foster the development of meaning in life, thereby influencing depressive symptoms through the proposed pathway.

Among various interventions, group counseling is widely used in psychology and education. It involves a professional facilitator guiding group members through strategies and techniques designed to enhance self-awareness, improve interpersonal relationships, and develop new behavioral patterns through interactions among members (23). Compared to individual counseling, group counseling offers the advantage of addressing shared psychological concerns more efficiently.

**Figure 1:**
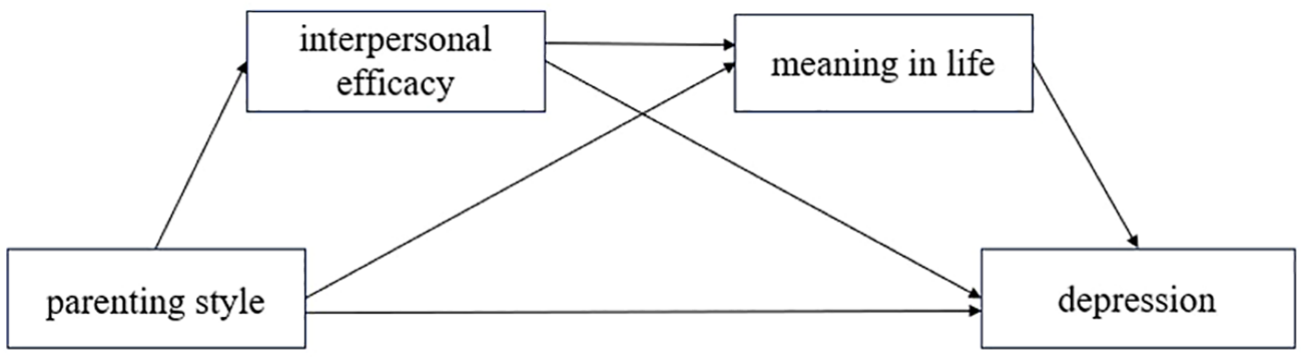
Hypothesized model for Study 1.

Building on these theoretical and practical considerations, the present study employed a two-stage design. First, a serial mediation model was tested to examine the relationships among parenting styles (positive and negative), interpersonal efficacy, meaning in life, and depression (**Figure 1**). It is important to note that alternative configurations of these variables are also plausible. Therefore, the proposed model represents one theoretically grounded pathway rather than a definitive causal mechanism. Second, a group counseling intervention targeting interpersonal efficacy was implemented to assess whether enhancing this resource is associated with changes in depressive symptoms. This phase aimed to provide preliminary experimental evidence for the proposed framework and offer insights for depression interventions among college students.

## 2 Study 1: chain mediation of interpersonal efficacy and meaning in life between parenting styles and depression

### 2.1 Materials and methods

#### 2.1.1 Participants

This study was conducted at a medical university in Guizhou Province, Southwest China. Guizhou is a multi-ethnic region with large Miao and Dong populations (24). The province also has a significant migrant worker population, and a high proportion of students come from left-behind families, which may influence parenting experiences and their retrospective reporting. Epidemiological data indicate that depressive symptoms are prevalent among college students in this region, with reported rates ranging from 20.2% to 37.6% (25, 26). These regional characteristics provide important context for interpreting the findings.

A total of 1,262 college students were recruited through cluster sampling. Participants completed both offline and online questionnaires. Data points exceeding three standard deviations from the mean were considered outliers, and 118 questionnaires were excluded, resulting in 1,144 valid responses (valid response rate: 90.6%).

#### 2.1.2 Measures

##### 2.1.2.1 Revision of the short-form Egna Minnen av Barndoms Uppfostran for Chinese

Developed by Arrindell et al. and revised by Jiang Jiang for the Chinese population, this version includes two subscales (father and mother), each comprising 21 items, for a total of 42 items. The questionnaire measures three dimensions: rejection, emotional warmth, and overprotection. A 4-point scale is used, ranging from 1 (‘Never’) to 4 (‘Always’), with Items 29 and 30 being reverse-scored. Higher scores reflect stronger perceptions of the corresponding parenting dimension (27). In this study, Cronbach’s α coefficients for the subscales ranged from 0.672 to 0.878. The overprotection subscales showed slightly lower internal consistency (father: α=0.67; mother: α=0.69), which is consistent with prior findings in Chinese samples (28). Since this study collected parenting style data through retrospective reports, recall bias may be a potential concern.

##### 2.1.2. 2 College students’ interpersonal efficacy scale

Developed by Xie Jing to measure individuals’ interpersonal efficacy levels, this scale comprises six dimensions: affinity efficacy, altruistic efficacy, communication efficacy, self-worth, emotional control efficacy, and self-impression efficacy. Each dimension contains six items, totaling 36 items. A 6-point scale is used, ranging from 1 (‘completely disagree’) to 6 (‘completely agree’). Some items are reverse-scored. Higher scores indicate greater interpersonal efficacy (16). In this study, the Cronbach’s α coefficient for this scale was 0.752.

##### 2.1.2.3 The meaning in life questionnaire

Developed by Steger et al. and revised into Chinese by Liu Sisi and Gan Yiqun, It is used to measure an individual’s sense of life meaning. The scale consists of 10 items across two dimensions: the presence of meaning and the search for meaning. It uses a 7-point scale ranging from 1 (‘Strongly Disagree’) to 7 (‘Strongly Agree’), with 9 items reverse-scored. Higher scores indicate greater levels of the corresponding dimension (19). In this study, the Cronbach’s α coefficient for this scale was 0.861.

##### 2.1.2.4 Center for epidemiological survey, depression scale

Developed by Radloff and revised by Chen Ziyan et al., this tool assesses an individual’s level of depression. It consists of 20 items scored on a 4-point scale ranging from 0 (‘Rarely, lasting less than one day’) to 3 (‘Most of the time, lasting 5 to 7 days’). The total score is the sum of all item scores, with higher scores indicating greater depression severity (29). In this study, the Cronbach’s α coefficient for this scale was 0.879.

#### 2.1.3 Analysis

Data were analyzed using SPSS 27.0. Pearson correlation analysis was conducted to examine the relationships among variables. Model 6 of the PROCESS macro was used to analyze the chained mediating effects of interpersonal efficacy and meaning in life on the relationship between parenting styles and depression.

### 2.2 Results

#### 2.2.1 Common method bias test

Common method bias was assessed using Harman’s single-factor test, which provides a preliminary evaluation. The results revealed 26 factors with eigenvalues greater than 1, with the first common factor explaining 14.92% of the variance, which is below the critical threshold of 40%. This suggests that common method bias is unlikely to significantly impact the findings.

#### 2.2.2 Correlation analysis

Pearson correlation analysis was conducted on parenting styles, interpersonal efficacy, meaning in life, and depression. The correlation matrix is presented in **Table 1**. The results indicate that positive parenting styles are positively correlated with interpersonal efficacy, meaning in life, and its sub-dimensions, while negatively correlated with depression. In contrast, negative parenting styles—parental rejection and overprotection—were negatively correlated with interpersonal efficacy, meaning in life, and its sub-dimensions, and positively correlated with depression (*p* < 0.01 for all correlations).

**Table 1:** Correlation analysis.

Variable	M ± SD	1	2	3	4	5	6	7	8
Parental Rejection ^M^	1.41 ± 0.41	1							
Parental Emotional Warmth ^M^	2.71 ± 0.56	-0.35**	1						
Parental Overprotection ^M^	1.97 ± 0.39	0.60**	-0.18**	1					
Interpersonal Efficacy ^T^	137.10 ± 11.94	-0.32**	0.39**	-0.24**	1				
the presence of meaning ^T^	22.60 ± 4.25	-0.23**	0.31**	-0.20**	0.48**	1			
the search for meaning ^T^	24.82 ± 4.47	-0.21**	0.26**	-0.09**	0.38**	0.51**	1		
Meaning in Life ^T^	47.42 ± 7.57	-0.26**	0.33**	-0.16**	0.49**	0.86**	0.88**	1	
Depression ^T^	15.00 ± 8.61	0.42**	-0.26**	0.34**	-0.44**	-0.32**	-0.17**	-0.28**	1

^M^ = Mean score; ^T^ = Total score. *indicates *p* < 0.05, **indicates *p* < 0.01, ***indicates *p* < 0.001; same applies below.

#### 2.2.3 Chain mediation analysis of interpersonal efficacy and sense of life meaning in the relationship between parenting styles and depression

##### 2.2.3.1 Chain analysis of interpersonal efficacy and meaning in life mediating the relationship between positive parenting styles and depression

This study examines the chain mediation effect model using positive parenting style as the independent variable, depression as the dependent variable, and interpersonal efficacy and meaning in life as mediating variables. Model 6 of the PROCESS macro was employed to analyze the mediating roles of interpersonal efficacy and meaning in life in the relationship between positive parenting style and depression.

**Figure 2:**
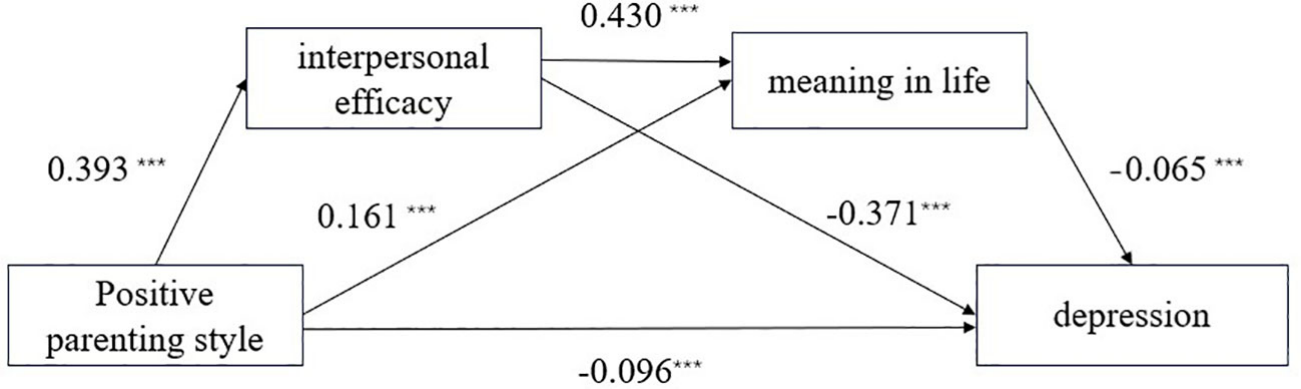
Chain mediation effect of interpersonal efficacy and meaning in life between positive parenting styles and depression. ***p<0.001.

The results of the chained mediation analysis are presented in **Table 2** and **Figure 2**. Positive parenting style had a positive predictive effect on both interpersonal efficacy and meaning in life (*β* = 0.393, *p* < 0.001; *β* = 0.161, *p* < 0.001), and a significant negative predictive effect on depression (*β* = -0.2636, *p* < 0.001). After simultaneously incorporating interpersonal efficacy and meaning in life into the model, the predictive effect of positive parenting on depression remained significant (*β* = -0.096, *p* < 0.001). Interpersonal efficacy positively predicted meaning in life (*β* = 0.430, *p* < 0.001), while meaning in life negatively predicted depression (*β*=−0.065, *p* < 0.05). The full model accounted for 20.7% of the variance in depressive symptoms (*R²* = 0.207), indicating a moderate effect size. These results are statistically consistent with a chain mediation model in which positive parenting is associated with depression through interpersonal efficacy and meaning in life.

**Table 2:** Chain mediation analysis model of interpersonal efficacy and meaning in life mediating the relationship between positive parenting and depression.

Item	Interpersonal Efficacy			Meaning in Life			Depression		
	*β*	t-value	p-value	*β*	t-value	p-value	*β*	t-value	p-value
Positive parenting style	0.393	14.441	<0.001	0.161	5.815	<0.001	-0.096	-3.309	0.001
Interpersonal Efficacy				0.430	15.583	<0.001	-0.371	-11.758	<0.001
Meaning in Life							-0.065	-2.116	0.035
*R²*		0.154			0.265			0.207	
F-value		208.540			205.696			99.442	
P-value		<0.001			<0.001			<0.001	

Using the Bootstrap method with a 95% confidence interval, a significance level of α=0.05, and 5000 resamples, an interval not containing 0 indicates a significant mediating effect. **Table 3** shows that the 95% confidence interval for interpersonal efficacy between positive parenting and depression is [-0.1785, -0.1159], which does not include 0, indicating a significant mediating effect. The 95% confidence interval for meaning in life between positive parenting and depression is [-0.0214, -0.0009], which does not include 0, indicating a significant mediating effect; which also does not include 0, indicating a significant mediating effect. Similarly, the 95% confidence interval for the chain mediation of interpersonal efficacy and meaning in life between positive parenting and depression is [-0.0220, -0.0010], which does not include 0, indicating a significant chain mediating effect. All path coefficients showed statistically significant differences.

**Table 3:** Testing the chained mediating effects of interpersonal efficacy and meaning in life between positive parenting and depression.

Item	Effect size	Boot SE	95% CI		Relative effect proportion (%)
Indirect Effect	-0.1673	0.0156	-0.1985	-0.1384	63.47%
Positive parenting style → Interpersonal efficacy → Depression	-0.1459	0.0158	-0.1785	-0.1159	55.35%
Positive parenting → Meaning in life → Depression	-0.0104	0.0053	-0.0214	-0.0009	3.95%
Positive parenting → Interpersonal efficacy →Meaning in life → Depression	-0.0110	0.0053	-0.0220	-0.0010	4.17%
Direct Effect	-0.0963	0.0291	-0.1534	-0.0392	36.53%
Total Effect	-0.2636	0.0285	-0.3196	-0.2076	100%

Path Analysis Results As shown in **Table 3**, positive parenting was associated with college students’ depression levels through interpersonal efficacy, with an indirect effect of -0.1459, accounting for 55.35% of the total effect. Positive parenting also mediates depression levels via meaning in life, with a mediating effect of -0.0104, accounting for 3.95% of the total effect. Furthermore, interpersonal efficacy and meaning in life exert a chain mediating effect between positive parenting and depression, with a mediating effect of -0.0110 accounting for 4.17% of the total effect.

##### 2.2.3.2 Mediated chain analysis of the relationship between interpersonal efficacy, the presence of meaning, negative parenting, and depression

Using negative parenting as the independent variable, depression as the dependent variable, and interpersonal efficacy and the presence of meaning as mediating variables, we tested the chained mediation effect model. Model 6 of the PROCESS macro was employed to analyze the chained mediating effects of interpersonal efficacy and the presence of meaning in the relationship between negative parenting and depression.

**Figure 3:**
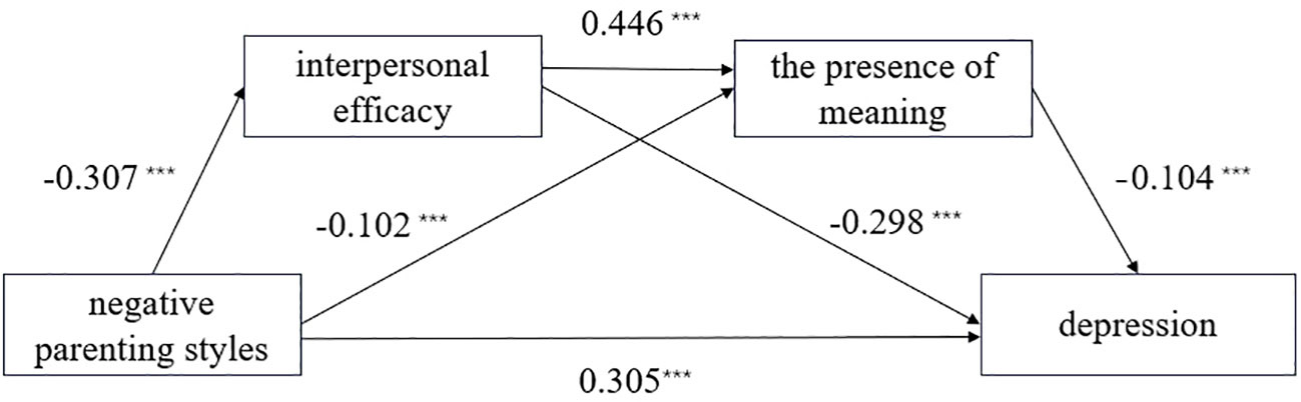
Chain mediation effect of interpersonal efficacy and the presence of meaning between negative parenting styles and depression. ***p<0.001.

The results of the chained mediation effect test are presented in **Table 4** and **Figure 3**. Negative parenting styles had a negative predictive effect on interpersonal efficacy and the presence of meaning (*β* = -0.307, *p* < 0.001; *β* = -0.102, *p* < 0.001), and a positive predictive effect on depression (*β* = 0.4216, *p* < 0.001).After simultaneously incorporating interpersonal efficacy and the presence of meaning into the model, the predictive effect of negative parenting on depression remained significant (*β* = 0.305, *p* < 0.001). Interpersonal efficacy positively predicted the presence of meaning (*β* = 0.446, *p* < 0.001), while the presence of meaning negatively predicted depression (*β*=−0.104, *p* < 0.001). The full model accounted for 29.3% of the variance in depressive symptoms (*R²* = 0.293), indicating a moderate effect size. These results are statistically consistent with a chain mediation model in which negative parenting is associated with depression through interpersonal efficacy and the presence of meaning.

**Table 4:** Chain mediated effect model of interpersonal efficacy and the presence of meaning in the relationship between negative parenting and depression.

Item	Interpersonal Efficacy			the presence of meaning			Depression		
	*β*	t-value	p-value	*β*	t-value	p-value	*β*	t-value	p-value
Negative parenting style	-0.307	-10.909	<0.001	-0.102	-3.754	<0.001	0.305	11.596	<0.001
Interpersonal Efficacy				0.446	16.437	<0.001	-0.298	-10.230	<0.001
the presence of meaning							-0.104	-3.654	<0.001
*R* ^2^		0.094			0.238			0.293	
F-value		119.001			177.866			157.715	
P-value		<0.001			<0.001			<0.001	

As shown in **Table 5**, the 95% confidence interval for interpersonal efficacy between negative parenting styles and depression is [0.0709, 0.1138], which does not include 0, indicating a significant mediating effect. The 95% confidence interval for the presence of meaning between negative parenting styles and depression is [0.0039, 0.0191], which also does not include 0, indicating a significant mediating effect. The 95% confidence interval for the chained mediating effect between interpersonal efficacy and the presence of meaning in the relationship between negative parenting styles and depression is [0.0065, 0.0233], which does not include 0, indicating a significant chained mediating effect. All path coefficients showed statistically significant differences.

**Table 5:** Testing the chained mediating effects of interpersonal efficacy and the presence of meaning between negative parenting styles and depression.

Item	Effect size	Boot SE	95% CI			Relative effect proportion (%)
Indirect Effect	0.1164	0.0118	0.0942	0.1401	27.61%	
Negative parenting style → Interpersonal efficacy → Depression	0.0914	0.0110	0.0709	0.1138	21.68%	
Negative parenting style →the presence of meaning → Depression	0.0106	0.0039	0.0039	0.0191	2.51%	
Negative parenting style → Interpersonal efficacy →the presence of meaning → Depression	0.0143	0.0043	0.0065	0.0233	3.39%	
Direct Effect	0.3052	0.0263	0.2536	0.3569	72.39%	
Total Effect	0.4216	0.0268	0.3690	0.4742	100%	

Path analysis results (**Table 5**) indicated that negative parenting was associated with college students’ depression levels through interpersonal efficacy, with an indirect effect of 0.0914 accounting for 21.68% of the total effect. Negative parenting styles were also associated with depression levels through the presence of meaning, with a mediating effect of 0.0106, accounting for 2.51% of the total effect. Furthermore, interpersonal efficacy and the presence of meaning exert a chain mediation effect between negative parenting styles and depression, with a mediating effect of 0.0143, accounting for 3.39% of the total effect.

### 2.3 Discussion

#### 2.3.1 Current status of depression

Referencing Radloff’s study, which identifies a score of 16 as the threshold for “possible depressive problems “ (29), the survey indicates that 41.96% of participants may experience depressive symptoms. This suggests that a significant proportion of college students are vulnerable to depressive emotions. Therefore, understanding the psychological processes associated with depressive symptoms is crucial.

#### 2.3.2 Chain mediation effect of interpersonal efficacy and meaning in life between parenting styles and depression

Study 1 found that parenting styles were associated with interpersonal efficacy, meaning in life, and depression in a manner consistent with a sequential mediation model. However, due to the cross-sectional design, these results cannot be interpreted as evidence of causal relationships. The observed associations may be bidirectional or reflect the influence of unmeasured confounding variables. Longitudinal studies are needed to examine the temporal ordering of these variables. If such variables are associated with both parenting styles and depressive symptoms, the observed associations could be either overestimated or underestimated. It is therefore possible that the true relationships differ from those reported here. Moreover, although Harman’s single-factor test suggested that common method bias was not a major concern, this test has limited sensitivity. Because all variables were measured at the same time point, the observed associations may still be inflated—an inherent limitation of cross-sectional designs. This limitation should be considered when interpreting the findings, as the true strength of the associations may be lower than reported. Additionally, the internal consistency of the overprotection subscale was slightly lower than that of other dimensions, which may reflect the multifaceted nature of parental control in the Chinese cultural context. To better understand the theoretical pathways and empirical evidence linking parenting styles to these psychological resources, the following sections draw on relevant theoretical perspectives and prior research. According to ecological systems theory, parents are individuals’ earliest and most influential interaction partners, and parenting styles constitute a critical microsystem that shapes psychological development (30). Positive parenting styles foster the development of a stable and confident self-concept and enhance individuals’ sense of competence in interpersonal interactions, thereby increasing interpersonal efficacy (17). In contrast, individuals exposed to negative parenting—characterized by rejection or overprotection—are more likely to develop negative evaluations of their social competence, resulting in lower interpersonal efficacy. This is consistent with findings that parental rejection is linked to depression through its association with children’s cognitive styles (31), supporting cognitive theories of depression (32).

Building on interpersonal efficacy, meaning in life plays a further role in the chained pathway. From the perspective of Maslow’s hierarchy of needs, higher interpersonal efficacy helps individuals fulfill their belongingness and esteem needs, which serve as important sources of meaning in life (33). Consistently, research has found that interpersonal competence significantly predicts meaning in life among Chinese college students, supporting the idea that positive interpersonal experiences facilitate the construction of meaning (34). In turn, a higher level of meaning in life promotes clearer life goals and a stronger value orientation, thereby reducing the risk of depression (35). This is also supported by a recent meta-analysis showing a moderate negative correlation between meaning in life and depression (36).

Beyond the overall mediating effects, differential roles emerged for the dimensions of meaning. Notably, the presence of meaning exerted a stable mediating effect between negative parenting and depression, whereas the search for meaning did not. This finding aligns with research showing that the presence of meaning is a stronger predictor of mental health than the search for meaning (37). The search for meaning may only exert its influence when it is successfully transformed into the presence of meaning (38)—a process that can be facilitated by positive family environments (39).

In summary, interpersonal efficacy and meaning in life jointly constitute a continuous psychological pathway linking parenting styles and depression. This chained mediation model integrates the roles of family environment, interpersonal functioning, and meaning construction, thereby enriching theoretical explanations of the mechanisms underlying depression among college students. Moreover, the findings suggest that intervention efforts should target parenting practices, with a focus on enhancing individuals’ interpersonal efficacy and fostering a stable sense of meaning in life to reduce the risk of depression.

Lastly, regarding the generalizability of these findings, while no studies have directly tested the full sequential mediation model across cultures, each individual pathway has received empirical support in multiple cultural contexts. Positive parenting predicts interpersonal efficacy in both Western and Asian samples (40). Interpersonal efficacy consistently predicts meaning in life across cultures (41, 42). Meta-analyses confirm that meaning in life protects against depression in diverse populations (43). Collectively, these findings suggest that the proposed model may operate similarly across cultural contexts, though direct cross-cultural comparisons are needed to confirm this hypothesis.

## 3 Study II: effects of group counseling intervention

### 3.1 Materials and methods

#### 3.1.1 Participants

Participants with low interpersonal efficacy and high depression scores (the top 27% based on questionnaire results from Study 1) (45), without a psychiatric history, and who voluntarily participated in group counseling, were randomly assigned to two groups by drawing lots. Group assignment was revealed only after baseline assessments were completed. According to G*Power 3.1 software, with a significance level of α=0.05, an effect size of 0.25, and 80% statistical power, the minimum required sample size was 28 participants. Considering potential participant attrition, this study initially recruited 35 participants, with 17 assigned to the experimental group and 18 to the control group. The experimental group received group psychological counseling, while the control group received no intervention. After the intervention, 3 participants dropped out of the experimental group and 4 from the control group, resulting in 14 participants in each group. The final distribution of participants is presented in **Table 6**.

**Table 6:** Distribution of demographic variables in the control and intervention groups.

Variable	Dimension	Intervention group (14 participants)	Control group (14 individuals)
Gender	Male	6	6
	Female	8	8
Place of Origin	Town	4	3
	Rural	10	11
Grade	Freshman	6	6
	Sophomore	6	6
	Junior	2	2
	Senior Year	0	0
Major	Liberal Arts	2	5
	Science and Engineering	6	6
	Arts and Physical Education	1	0
	Medicine	6	3

#### 3.1.2 Research design

The primary purpose of Study 2 was to examine whether interpersonal efficacy could be enhanced through a group counseling intervention. A secondary aim was to explore whether changes in interpersonal efficacy might be associated with depressive symptoms, as suggested by the sequential mediation model in Study 1. A 3 (time: pretest, posttest, 3-month follow-up) × 2 (group: experimental, control) mixed-design was employed, with gender included as a covariate to control for potential confounding effects. Questionnaires were administered to participants in both groups at three time points: before the intervention, immediately after the intervention, and three months after the intervention, which constituted the within-subjects factor. The experimental group participated in seven weekly group counseling sessions, each lasting approximately 90 minutes, while the control group received no intervention. The specific group intervention protocol is shown in **Table 7**. Group membership served as the between-subjects factor. A non-active control group was used due to resource limitations. Participants in the experimental group were aware of their group assignment. Participants in the control group were informed that they were assigned to a non-intervention group but were not given detailed information about the study purpose. The facilitator was aware of group assignment. Data analysts were not blinded to group assignment, as the same researcher performed both the intervention and the statistical analysis. All sessions were conducted by the same trained facilitator, who had graduate-level training in psychology and previous experience leading group activities. The intervention followed a structured program framework that specified session themes and core activities to ensure overall consistency. At the same time, limited adaptations were made to accommodate group dynamics and participants’ feedback while preserving the predefined session objectives. Although no formal fidelity checklist was employed, adherence to the predefined core components and session objectives was maintained throughout the intervention. This study was approved by the Ethics Review Committee of Guizhou Medical University (Approval No. 401).

**Table 7:** Intervention group counseling program.

Unit name	Unit counseling objective	Unit activity flow
1. Initial Familiarization	Introduction to group counseling, informed consent, and initial relationship building	1.Introduction to research design and basic group counseling knowledge, signing group contract (15 min)
		2.Body scan relaxation exercise (10 min)
		3.Wind blows (15 min) snow balling (10 min)
		4.OH Cards self-introduction (15 min)
		5.Interpersonal self-portrait (20 min)
2. Impression Management	Enhance impression management efficacy by understanding how others perceive you, and develop impression management strategies	1.Relaxation training (10 min) musical chairs (15 min)lucky pillow squat (10 min)
		2.My Impression (15 min)
		3.Anonymous Feedback (30 min)
		4.Sharing impression management strategies and closing reflections (15 min)
3. Connecting with Others	Enhance affiliative efficacy; learn to accept and praise others through interaction	1.Relaxation exercise (10 min) unlocking the chain (10 min)
		2.Relay drawing: sketch your imagined home (40 min)
		3.Strengths bombardment (20 min)
		4.Sharing moments of connection with close friends and groups (10 min)

#### 3.1.3 Research instruments

##### 3.1.3.1 Questionnaires

The College Students’ Interpersonal Efficacy Scale and the Center for Epidemiologic Studies Depression Scale used in this study were the same as those employed in Study 1.

##### 3.1.3.2 Group counseling program

The intervention for the experimental group employed a self-designed group counseling program. The activities were structured around the six dimensions of interpersonal efficacy: affiliative efficacy, self-impression efficacy, benefit-other efficacy, communication efficacy, mood-control efficacy, and self-value efficacy. Affiliative efficacy refers to individuals’ perceived confidence in initiating interpersonal contact, developing intimacy, and maintaining close relationships. Self-impression efficacy reflects individuals’ perceived ability to form and manage favorable impressions of themselves in social interactions. Benefit-other efficacy assesses individuals’ confidence in helping others and responding empathetically to others’ needs. Connection efficacy refers to individuals’ perceived ability to establish and maintain stable interpersonal connections. Mood-control efficacy reflects individuals’ perceived ability to regulate emotional states and minimize the negative impact of emotions on interpersonal interactions. Self-value efficacy represents individuals’ stable self-evaluation of their importance and value within interpersonal relationships. The detailed content of the group counseling program is presented in **Table 7**.

##### 3.1.3.3 Group counseling session feedback form

A self-developed Group Counseling Session Feedback Form was used to collect participants’ feedback on each group counseling session. Based on members’ feedback, the facilitator evaluated the effectiveness of each session and adjusted subsequent activities accordingly to optimize the intervention process. The feedback form primarily covered participants’ overall evaluation of the group activities, assessment of the facilitator, personal takeaways from the session, perceived strengths of the activity design, suggestions for improvement, and other relevant comments.

##### 3.1.3.4 Group member evaluation form

A self-developed Group Member Evaluation Form was completed by participants upon finishing the intervention. Participants were guided through structured reflection and self-assessment to consolidate their intervention experiences and perceived personal growth. Afterward, the completed forms were shared and discussed within the group.

#### 3.1.4 Statistical analysis

This study analyzed data from participants who completed all intervention sessions and assessments (per-protocol analysis). Missing data were handled by excluding participants who did not complete all intervention sessions and assessments. After organizing the repeated-measures data from the three time points for both the experimental and control groups, SPSS version 27.0 was used to perform repeated-measures analyses of variance. Between-group main effects, within-group main effects, and group × time interaction effects were examined for all outcome variables across the three measurement occasions.

### 3.2 Results

#### 3.2.1 Homogeneity test

Pre-intervention analyses indicated no statistically significant differences between the experimental and control groups in interpersonal efficacy or depression scores (*p* = 0.647, *p* = 0.325, **Table 8**), suggesting that the assumption of baseline homogeneity was tenable.

**Table 8:** Comparison of scale scores between groups at different intervention timepoints.

Scale	Group	Pre-intervention	After intervention	3 Months post-intervention	*p*_12_ *(d)*	*p*_13_ *(d)*	*p*_23_ *(d)*
Interpersonal efficacy	Experimental Group	124.57 ± 3.06	137.21 ± 12.99	130.43 ± 7.98	<0.001 (1.49)	0.022 (0.94)	0.039 (-0.62)
	Control group	123.57 ± 7.53	126.71 ± 10.11	125.07 ± 8.42	0.813 (0.23)	1.000 (0.17)	1.000 (-0.06)
	*p(d)*	0.647(0.17)	0.026(0.90)	0.097(0.65)			
Depression	Experimental group	22.93 ± 5.92	19.29 ± 7.14	18.79 ± 9.37			
	Control Group	25.29 ± 6.50	19.43 ± 7.72	21.71 ± 8.32			
	*p(d)*	0.325(−0.38)					

*P*_12_ and *P*_13_ represent the P-values comparing pre-intervention to post-intervention and post-intervention to 3 months post-intervention, respectively; *P*_23_ represents the P-value comparing post-intervention to 3 months post-intervention; Cohen’s d in parentheses. interpretation: d≥0.2=small effect, d≥0.5=medium effect, d≥0.8 =large effect.

#### 3.2.2 Effect of group counseling intervention on interpersonal efficacy

Mauchly’s test of sphericity was not significant (*p*>0.05), indicating that the assumption of sphericity was met. Accordingly, repeated-measures ANOVA was conducted to examine interpersonal efficacy and depression, with the results presented in **Table 9**.

**Table 9:** Results of repeated measures.

Scale	Effect	*F*	*df*	*p*	*η_p_^2^*
Interpersonal Efficacy	Group	4.337^*^	1, 26	0.048	0.148
	Time Point	0.689	2, 52	0.507	0.027
	Interaction Effect	3.717^*^	2, 52	0.031	0.129
Depression	Group	0.667	1, 26	0.422	0.026
	Time Point	2.986	2, 52	0.060	0.107
	Interaction Effect	0.424	2, 52	0.656	0.017

Partial eta-squared (*η_p_^2^*) was interpreted using Cohen’s (1988) guidelines: 0.01=small effect, 0.06=medium effect, 0.14=large effect (44).

For interpersonal efficacy scores, the main effect of group was significant, *F*(1,26)=4.337, *p* = 0.048, *η_p_^2^* = 0.148, indicating a moderate effect size, with the intervention group showing a higher overall mean score than the control group by 5.62 points (*p* = 0.048, **Table 10**), Cohen’s *d* is 2.94, indicating a large effect size and a significant intervention effect. Additionally, the group × time point interaction was significant, *F*(2,52)=3.717, *p* = 0.031, *η_p_^2^* = 0.129, suggesting differential change patterns across time between the two groups. The magnitude of this interaction effect was also moderate.

**Table 10:** Comparison of interpersonal efficacy scores across different intervention timelines between groups.

Scale	Group	Pre-intervention	After intervention	3 Months post-intervention	Total	Difference	*p(d)*
Interpersonal efficacy	Experimental group	124.57 ± 3.06	137.21 ± 12.99	130.43 ± 7.98	130.74 ± 1.91	5.62	0.048 (2.94)
	Control group	123.57 ± 7.53	126.71 ± 10.11	125.07 ± 8.42	125.12 ± 1.91		

Cohen’s d in parentheses. interpretation: d≥0.2=small effect, d≥0.5=medium effect, d≥0.8=large effect.

**Figure 4:**
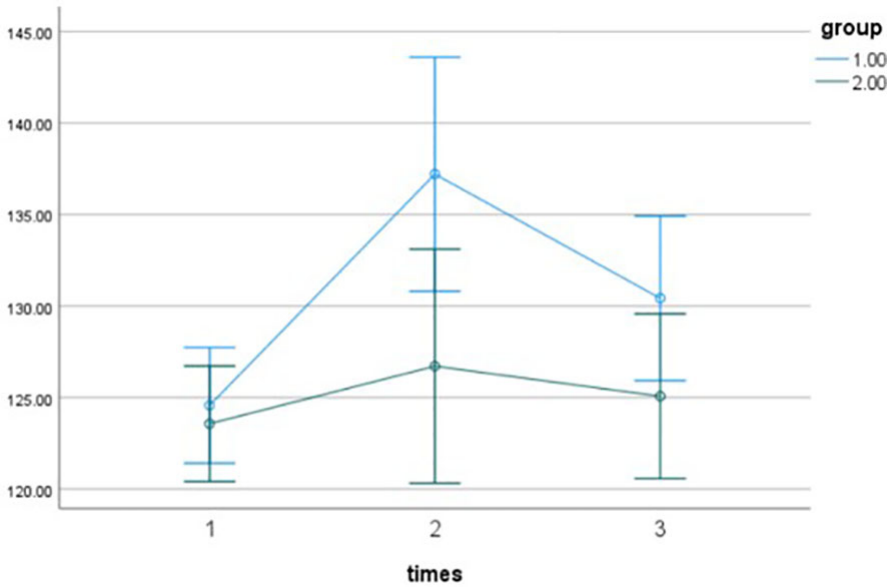
Interaction plot of interpersonal efficacy.

To further explore this interaction, simple effects analyses with a Bonferroni correction were conducted, and the results are presented in **Table 8**. First, when comparing the experimental and control groups at each time point, no significant between-group difference was observed at pre-intervention (*p* = 0.647), satisfying the homogeneity of groups assumption. Post-intervention, the difference between the two groups was statistically significant (*p* = 0.026), with the experimental group scoring higher than the control group. At 3 months post-intervention, the difference in scores between the two groups was no longer statistically significant (*p* = 0.097). Second, within-group comparisons across time points revealed significant changes in the experimental group (**Table 8**). Post-intervention scores were higher than pre-intervention scores (*p*_12_<0.001). Although scores at 3 months post-intervention were lower than immediately post-intervention (*p*_23_ = 0.039), t they remained significantly higher than pre-intervention scores (*p*_13_ = 0.022). In the control group, no statistically significant differences were observed between the three time points (all *p*>0.05). Based on the results of the simple effects analysis, the pattern of change in interpersonal efficacy is as follows: The experimental group’s scores significantly increased after the intervention. Although they decreased slightly during the follow-up period compared to the post-intervention level, they remained higher than the baseline. In contrast, the control group’s scores remained stable at all time points. This dynamic trend is illustrated in **Figure 4**.

#### 3.2.3 Effects of group counseling intervention on depression

**Figure 5:**
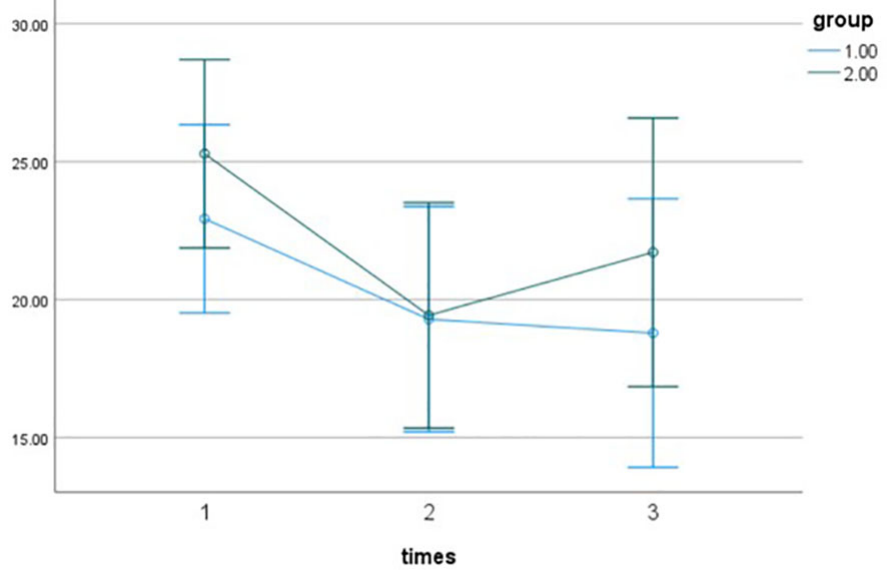
Interaction plot of depression.

Regarding depression scores (**Table 9**), the main effect of group assignment was not significant, *F*(1,26)=0.667, *p* = 0.422, *η_p_^2^* = 0.026; The main effect of time point was also not significant, *F*(2,52)=2.986, *p* = 0.060, *η_p_^2^* = 0.107, Additionally, the interaction between group and time point was not significant, *F*(2,52)=0.424, *p* = 0.656, *η_p_^2^* = 0.017. Descriptive means and change trends for depression scores are shown in **Figure 5**.

### 3.3 Discussion

#### 3.3.1 Effects of group counseling intervention on interpersonal efficacy

The experimental group demonstrated significantly higher overall levels of interpersonal efficacy than the control group and showed a distinct change pattern: scores peaked post-intervention, declined slightly at follow-up, but remained above baseline. Several factors may explain these effects.

First, the group setting itself provided a supportive interpersonal environment conducive to enhancing interpersonal efficacy (23). Second, each session translated abstract interpersonal efficacy into concrete sub-dimensions, allowing members to experience micro-level success (46). Third, the structured format promoted engagement through body scan relaxation, icebreaker games, thematic activities (e.g., anonymous feedback), homework assignments, and reflective summaries. These components collectively enhanced self-awareness and efficacy, with unit feedback forms allowing real-time adjustments (23, 47–49).

The regression observed in the experimental group during the follow-up period may stem from insufficient transfer of skills into daily life. Changes facilitated by group counseling require reinforcement through regular practice. However, this study lacked reinforcement components during the follow-up phase, and the frequency of homework assignments and participant compliance during the intervention period were limited. These factors may have hindered the full generalization of the acquired skills (50). This phenomenon is also commonly observed in other short-term intervention studies (51, 52), highlighting the importance of designing effective maintenance strategies. Future practice should focus on extending intervention effects through more systematic post-session practice and ongoing support.

It is encouraging that follow-up scores were significantly higher than pre-intervention scores, demonstrating that the intervention helped participants elevate their baseline levels of interpersonal efficacy. In stark contrast, the control group’s data remained stable throughout the study, clearly indicating that the observed intervention effects were attributable to the group counseling itself, rather than non-specific factors such as the passage of time or testing experience.

In conclusion, this group counseling program effectively enhanced participants’ interpersonal efficacy and achieved partial long-term maintenance. However, to sustain the intervention effects beyond the active period, future interventions should refine strategies for skill generalization. Specifically, strategies such as monthly booster sessions or the creation of digital support groups (e.g., via WhatsApp or WeChat) could be effective in reinforcing skills learned during the intervention and maintaining social support over time.

#### 3.3.2 Effects of group counseling intervention on depression

Statistical analysis revealed no significant main effects or interactions for depression levels across groups or time points, indicating that the group counseling intervention focused on enhancing interpersonal efficacy did not produce statistically significant improvements in depression. It is important to note that this null finding was not due to mild baseline symptom levels. Participants were drawn from the top 27% of depression scores in the preliminary screening, and baseline scores in both groups were in the moderate range (21–25 points; **Table 8**), indicating clear emotional distress and sufficient room for improvement, making them suitable candidates for the intervention. Most importantly, Study 1 demonstrated that interpersonal efficacy and meaning in life sequentially mediated the relationship between parenting styles and depression. In the positive parenting model, the chain mediation effect accounted for 4.17% of the total effect, while interpersonal efficacy alone accounted for 55.35% (**Table 3**). In the negative parenting model, these effects were 3.39% and 21.68%, respectively (**Table 5**). These findings suggest that interpersonal efficacy may be associated with depression both directly and indirectly through meaning in life.

The present intervention successfully enhanced interpersonal efficacy with a large effect size (*d* = 1.49) but did not target meaning in life. This may explain why gains in interpersonal efficacy did not translate into significant reductions in depressive symptoms. Future research could consider multi-component interventions that target both interpersonal efficacy and meaning in life to assess their feasibility and effectiveness (53). Several factors may explain this null finding. First, the small final sample size (n=28) may have reduced the ability to detect significant between-group differences (54). Second, due to the nature of the intervention, participants and the facilitator were aware of group assignment, and outcome assessors were not blinded. This lack of blinding may introduce performance and detection bias. Specifically, participants in the experimental group may have altered their behavior due to awareness of being observed, and the facilitator may have inadvertently provided differential attention. Additionally, the control group received no intervention, which does not allow us to rule out non-specific effects such as expectancy or attention. Participants in the experimental group may have experienced improvements simply due to the attention received during the intervention, rather than the specific content of the program. Such non-specific factors could potentially obscure the true effects of the intervention (55). However, if such non-specific effects were the main driver of change, depressive symptoms would likely have also improved, which they did not. This pattern suggests that non-specific factors alone are unlikely to fully account for the observed improvements in interpersonal efficacy. Nevertheless, the absence of an active control group remains a limitation, and future studies should include one to further isolate the specific effects of the intervention content. Third, heterogeneity among participants—such as differences in life circumstances, personality traits, and skill acquisition—may have affected the timing and magnitude of symptom improvement, a common issue in group interventions (23). Finally, depressive symptoms are influenced by complex interactions of psychological and contextual factors, such as family pressure, academic demands, and lack of social support. Even when internal resources improve, persistent external stressors may limit measurable symptom changes (56). Given these limitations, the findings should be considered preliminary and the certainty of evidence for effects on depressive symptoms remains limited.

In interpreting these results, it is important to recognize that improvements in interpersonal efficacy represent an intermediate outcome, reflecting successful activation of the intervention target, rather than direct clinical improvement in depressive symptoms.

## 4 General discussion

This study examined the proposed theoretical framework through cross-sectional modeling in Study 1 and a preliminary intervention test in Study 2. The serial mediation findings indicated that the relationships among parenting styles, interpersonal efficacy, meaning in life, and depressive symptoms were statistically consistent with a chain mediation model. Study 2 focused on evaluating a key component within this framework, and the results showed that group counseling significantly improved interpersonal efficacy with large effect sizes. However, despite these substantial gains in interpersonal efficacy, depressive symptoms did not show significant between-group differences. It is important to note that improvements in interpersonal efficacy represent an intermediate outcome, reflecting successful activation of the intervention target, rather than direct clinical improvement in depressive symptoms. Given the limitations of sample size, risk of bias, and imprecision, the findings should be considered preliminary and the certainty of evidence for effects on depressive symptoms remains limited. Future well-designed randomized controlled trials with adequate sample sizes, prespecified primary and secondary outcomes, and active comparators where feasible are needed to strengthen internal validity. Essential methodological improvements would include intention-to-treat analyses, transparent reporting of allocation concealment, appropriate handling of missing data, and longer follow-up periods. In addition, the sequential mediation model should ideally be tested in longitudinal or experimental designs capable of establishing temporal precedence, rather than inferred from cross-sectional mediation analyses alone. Beyond methodological refinements, future research could also explore whether combining interventions that target interpersonal efficacy with strategies to foster meaning in life might yield stronger effects on depressive symptoms. Interventions may also benefit from addressing contextual factors such as environmental stressors or social support and conducting subgroup analyses to identify for whom such approaches are most effective. Promoting skill generalization through follow-up strategies could help sustain intervention effects over time. Collectively, these efforts will facilitate the transition from preliminary pathway validation to more robust and meaningful symptom improvement.

## Data Availability

The raw data supporting the conclusions of this article will be made available by the authors, without undue reservation.
